# Impact of tumor-treating fields on the survival of Japanese patients with newly diagnosed glioblastoma: A multicenter, retrospective cohort study

**DOI:** 10.1093/noajnl/vdae176

**Published:** 2024-11-28

**Authors:** Masayuki Kanamori, Shunsuke Tsuzuki, Ichiyo Shibahara, Kuniaki Saito, Yoshiteru Shimoda, Kazuhiro Tanaka, Shigeru Yamaguchi, Manabu Natsumeda, Tomoo Matsutani, Mitsuto Hanihara, Mitsutoshi Nakada, Jun-Ichiro Kuroda, Masahide Matsuda, Koji Yoshimoto, Ushio Yonezawa, Yukihiko Sonoda, Koji Takano, Hajime Yonezawa, Yoshihiro Otani, Yukiko Nakahara, Masashi Uchida, Masahiro Nonaka, Yohei Mineharu, Yohei Kitamura, Shinji Yamashita, Takahiro Yamauchi, Yohei Miyake, Shoichi Deguchi, Takaaki Beppu, Kaoru Tamura, Shinichiro Koizumi, Yuichi Hirose, Kenichiro Asano, Ryo Hiruta, Manabu Kinoshita, Keisuke Miyake, Noriyuki Nakayama, Akihiro Inoue, Takahiro Ono, Takahiro Sasaki, Yukinori Akiyama, Shinjiro Fukami, Atsuo Yoshino, Yu Kawanishi, Taku Asanome, Takuhiro Yamaguchi, Masamichi Takahashi, Fumiyuki Yamasaki, Yoshiki Arakawa, Yoshitaka Narita

**Affiliations:** Department of Neurosurgery, Tohoku University Graduate School of Medicine, Sendai, Japan; Department of Neurosurgery, Tokyo Women’s Medical University, Tokyo, Japan; Department of Neurosurgery, Kitasato University School of Medicine, Sagamihara, Japan; Department of Neurosurgery, Kyorin University Facility of Medicine, Tokyo, Japan; Department of Neurosurgery, Tohoku University Graduate School of Medicine, Sendai, Japan; Department of Neurosurgery, Kobe University Graduate School of Medicine, Kobe, Japan; Department of Neurosurgery, Faculty of Medicine, Hokkaido University, Sapporo, Japan; Department of Neurosurgery, Brain Research Institute, Niigata University, Niigata, Japan; Department of Neurological Surgery, Graduate School of Medicine, Chiba University, Chiba, Japan; Department of Neurosurgery, University of Yamanashi, Yamanashi, Japan; Department of Neurosurgery, Kanazawa University, Kanazawa, Japan; Department of Neurosurgery, Faculty of Life Sciences, Kumamoto University, Kumamoto, Japan; Department of Neurosurgery, Institute of Medicine, University of Tsukuba, Tsukuba, Japan; Department of Neurosurgery, Graduate School of Medical Sciences, Kyushu University, Fukuoka, Japan; Department of Neurosurgery, Graduate School of Biomedical and Health Sciences, Hiroshima University, Hiroshima, Japan; Department of Neurosurgery, Faculty of Medicine, Yamagata University, Yamagata, Japan; Department of Neurosurgery, Osaka International Cancer Institute, Osaka, Japan; Department of Neurosurgery, Graduate School of Medical and Dental Sciences, Kagoshima University, Kagoshima, Japan; Department of Neurological Surgery, Okayama University Graduate School of Medicine, Dentistry and Pharmaceutical Sciences, Okayama, Japan; Department of Neurosurgery, Faculty of Medicine, Saga University, Saga, Japan; Department of Neurosurgery, St. Marianna University School of Medicine, Kawasaki, Kanagawa, Japan; Department of Neurosurgery, Kansai Medical University, Hirakata, Japan; Department of Neurosurgery, Graduate School of Medicine, Kyoto University, Kyoto, Japan; Department of Neurosurgery, Keio University School of Medicine, Tokyo, Japan; Division of Neurosurgery, Department of Clinical Neuroscience, Faculty of Medicine, University of Miyazaki, Miyazaki, Japan; Division of Medicine, Department of Neurosurgery, Faculty of Medical Sciences, University of Fukui, Fukui, Japan; Department of Neurosurgery, Graduate School of Medicine, Yokohama City University, Yokohama, Japan; Division of Neurosurgery, Shizuoka Cancer Center, Nagaizumi, Japan; Department of Neurosurgery, Iwate Medical University, Shiwa, Japan; Department of Neurosurgery, Tokyo Medical and Dental University, Tokyo, Japan; Department of Neurosurgery, Hamamatsu University School of Medicine, Hamamatsu, Japan; Department of Neurosurgery, Fujita Health University, Toyoake, Japan; Department of Neurosurgery, Hirosaki University Graduate School of Medicine, Hirosaki, Japan; Department of Neurosurgery, Fukushima Medical University, Fukushima, Japan; Department of Neurosurgery, Asahikawa Medical University, Asahikawa, Hokkaido, Japan; Department of Neurological Surgery, Faculty of Medicine, Kagawa University, Kagawa, Japan; Department of Neurosurgery, Graduate School of Medicine, Gifu University, Gifu, Japan; Department of Neurosurgery, Ehime University School of Medicine, Toon, Japan; Department of Neurosurgery, Akita University Graduate School of Medicine, Akita, Japan; Department of Neurological Surgery, School of Medicine, Wakayama Medical University, Wakayama, Japan; Department of Neurosurgery, Sapporo Medical University, Sapporo, Japan; Department of Neurosurgery, Tokyo Medical University, Tokyo, Japan; Department of Neurological Surgery, Nihon University School of Medicine, Tokyo, Japan; Department of Neurosurgery, Kochi Medical School, Kochi University, Kochi, Japan; Department of Neurosurgery, Brain Tumor Center, Nakamura Memorial Hospital, Sapporo, Japan; Division of Biostatistics, Tohoku University Graduate School of Medicine, Sendai, Japan; Department of Neurosurgery and Neuro-Oncology, National Cancer Center Hospital, Tokyo, Japan; Department of Neurosurgery, Graduate School of Biomedical and Health Sciences, Hiroshima University, Hiroshima, Japan; Department of Neurosurgery, Graduate School of Medicine, Kyoto University, Kyoto, Japan; Department of Neurosurgery and Neuro-Oncology, National Cancer Center Hospital, Tokyo, Japan

**Keywords:** Asian population, determinant of use, glioblastoma, survival, tumor-treating fields

## Abstract

**Background:**

The EF-14 clinical trial demonstrated the safety and efficacy of tumor-treating fields (TTFields) for newly diagnosed glioblastoma. This study aimed to clarify the current status, safety, and efficacy of TTFields in Japanese patients who meet the EF-14 inclusion criteria.

**Methods:**

This was a multicenter retrospective cohort study. Background, treatment, and outcome data of patients who satisfied the inclusion criteria of the EF-14 trial were collected from 45 institutions across Japan. The rate, determinants, and current status of TTField use, including its safety and efficacy in terms of progression and survival, were retrospectively investigated. This study was conducted in accordance with the STROBE checklist.

**Results:**

Among the 607 patients enrolled, 70 were excluded due to progressive disease during radiation and temozolomide therapy, age > 80 years old, and Karnofsky Performance Status score of <70. Among the remaining 537 patients, 210 (39%) underwent TTField treatment. Multivariate analysis revealed younger age and spouse as a caregiver as significant factors for TTField use. The compliance rate of TTField use exceeded 75% in 60% of patients, with a median TTField usage duration of 11 months. Skin disorders requiring medical treatment occurred in 56% of patients. Multivariate Cox proportional hazards analysis in the whole series and propensity score-matched analysis revealed that TTField use was not a prognostic factor for progression-free survival (PFS) or overall survival (OS).

**Conclusions:**

TTField use did not have a substantial effect on either PFS or OS in Japanese patients with glioblastoma, despite compliance rates comparable to those observed in the EF-14.

## Lay Summary

Glioblastoma is a very aggressive brain tumor. Tumor-treating fields (TTFields) use electric fields to interfere with tumor growth and have been shown to help patients with glioblastoma live longer when they are combined with surgery, chemotherapy, and radiation. The authors of this study wanted to better understand the use of TTFields in Japan. To do this, they collected and combined data from 45 different Japanese institutions. Their results showed that 210 of the 607 patients with newly diagnosed glioblastoma used TTFields. Younger patients and patients with a supportive caregiver were more likely to use the treatment. The treatment used by patients for 11 months, on average. A total of 56% of patients developed skin problems from treatment that required medical attention. Their results showed that the use of TTFields did not increase how long patients lived compared to those who did not use the treatment.

**Key Points**

- Effect of tumor-treating fields (TTFields) on survival was analyzed based on data from 537 Japanese patients.
- Cox proportional hazards model and propensity score-matched analysis were used.
- TTField use did not show a substantial benefit for newly diagnosed glioblastomas.

**Importance of the Study**

The EF-14 clinical trial has demonstrated the safety and efficacy of tumor-treating fields (TTFields) for newly diagnosed glioblastomas. However, the efficacy of TTFields remains unclear in Asian populations. Based on data obtained from 45 institutions across Japan, this study presents data on the current status of TTField usage and its efficacy in patients satisfying the EF-14 inclusion criteria. Notably, younger age and spouse as a caregiver were identified as significant factors for TTField use. Despite excellent compliance with TTField use, this multi-institutional study did not demonstrate a substantial impact of TTFields on progression-free survival or overall survival among patients newly diagnosed with glioblastoma. Although the actual mechanism remains unclear, this result can be attributed to differences in TTField sensitivity across different races and patient backgrounds.

The standard treatment strategies for glioblastoma (GB) include surgical resection, radiation therapy, and chemotherapy with temozolomide (TMZ).^1^ Recently, studies have confirmed the efficacy of carmustine wafer implantation^2–4^ and photodynamic therapy in prolonging the survival of patients with GB,^5^ leading to their approval in Japan. However, limited evidence is available regarding their use as standard therapy.

Besides the aforementioned therapies, tumor-treating fields (TTFields) have been confirmed to be safe and effective for newly diagnosed and recurrent GB in randomized clinical trials. In 2012, a randomized trial (EF-11) comparing TTFields with physician’s choice chemotherapy for recurrent GB showed that patients treated with TTFields had survival times comparable to those who received chemotherapy but showed superior health-related quality of life and subjective symptoms.^6^ In addition, a randomized study on newly diagnosed GB (EF-14) was conducted to compare radiation and TMZ with radiation and TMZ plus TTFields. In the mentioned study, TTFields were started at the same time as adjuvant TMZ and continued until the second recurrence or up to 2 years. The addition of TTFields significantly improved progression-free survival (PFS) and overall survival (OS).^7^ The excellent results of the aforementioned trial resulted in the approval of TTFields in Japan by December 2017 (https://www.mhlw.go.jp/web/t_doc?dataId=00tc3052&dataType=1&pageNo=3). However, owing to various factors, such as the need to carry a battery, constantly wear electrodes, and shave their hair, TTFields have not yet been fully accepted as a standard treatment. Socially, the TTField device requires a caregiver for wearing and managing the device; moreover, this device interferes with the social activities of the patients. The EF-14 trial was an open-label study with no sham control. Due to the lack of other supportive evidence, physicians were reluctant to recommend TTFields to their patients. In addition, limited understanding of the mechanism and insights about the predictors of individual benefit^8^ were inhibitory factors for physicians to recommend TTFields. In addition to these real-world obstacles, the efficiency of TTFields in Japanese patients remains unclear, given that only 5.9% of the patients included in the EF-14 trial were Asian.^7^ Several reports have demonstrated differences in the prognosis of GB between Asian and Caucasian populations.^9–11^ Survival data of patients with GB collected from the Surveillance, Epidemiology, and End Results database and clinical trials in Japan suggest that Asian patients with GB have a better prognosis than Caucasian patients with GB.^9–11^ This difference can be partially attributed to the difference in the proportion of GB with epidermal growth factor receptor (*EGFR*) gene amplification.^10^ Therefore, it remains unclear whether the results of the EF-14 trial can be applied to Asian GB. So far, few studies have demonstrated the efficacy of TTFields in Asian populations. The EF-14 trial showed that TTFields prolonged the survival of 39 Korean patients,^12^ whereas a previous single-center retrospective study from China reported that TTFields did not improve survival despite having the highest compliance rate.^13^

In this context, the present multicenter collaborative study aimed to clarify the efficacy of TTFields on survival as the primary endpoint, and to assess the usage rate and determinants of TTField use and safety as secondary endpoints, in Japanese patients who met the EF-14 inclusion criteria.

## Materials and Methods

### Ethics

This study was approved by the Institutional Ethical Committee of Tohoku University School of Medicine (2022-1-345), and participants were given the option to opt out of this study.

### Patients

This retrospective cohort study was conducted at 45 institutions throughout Japan. All participating institutions are academic centers. Of these, 42 and 3 are core and branch hospitals, respectively, of the Board Certification System adopted by the Japan Neurosurgical Society.

The inclusion and exclusion criteria were set according to those described in the EF-14 trial. All patients, including TTField users and nonusers, were registered to elucidate the current status of the use of TTFields and determine its efficacy. In brief, we included patients aged 18–80 years who had histologically confirmed and newly diagnosed GB according to the WHO Classification, revised 4th edition,^14^ and had a Karnofsky Performance Status (KPS) score of at least 70 before the start of adjuvant TMZ. Those who received carmustine wafers or photodynamic therapy were also included. Patients with progressive disease after radiation and TMZ based on the Macdonald criteria^15^ before adjuvant TMZ, those with infratentorial location and evidence of increased intracranial pressure, and those who received concomitant bevacizumab with radiation and TMZ were excluded. The provision of information regarding TTFields to patients was decided according to the practical guide for appropriate use of TTFields of the Japanese Society of Neuro-oncology at all institutions, and patients ultimately decided whether to undergo treatment with TTFields.

### Data Collection

We collected data from patients who completed radiation and TMZ from the date of TTField availability at their institution to June 2020 to ensure a 2-year follow-up period until June 2022. The following information was collected for all patients who satisfied the inclusion criteria: patient background characteristics, tumor-related characteristics, and treatment-related characteristics. Patient background characteristics included age; sex; employment status before disease onset; presence of caregivers before initial treatment; neurological symptoms; KPS; consciousness state defined by the Japan Coma Scale (JCS; 0 = Alert, 1 = Almost fully alert, 2 = Disorientation to time or place; 3 = Unable to say own name or date of birth)^16^; and presence of focal symptoms, such as paralysis and aphasia, before adjuvant TMZ. Tumor-related characteristics included tumor location, histological diagnosis, methylation status in the promoter of the O6-methylguanine DNA methyltransferase (*MGMT*) gene, and isocitrate dehydrogenase (*IDH*) gene mutation status based on an institutional diagnosis. Treatment-related characteristics included the extent of resection, residual enhancing lesion before adjuvant TMZ, use of carmustine wafer implantation and photodynamic therapy, and cycle number of adjuvant TMZ. During data collection, the extent of resection was classified into biopsy, residual measurable or unmeasurable enhancement, and no residual enhanced lesion. We defined a lesion with 2 perpendicular diameters of at least 10 mm as a “measurable” residual enhanced lesion.^16^ A residual lesion before adjuvant TMZ indicates an enhanced lesion, except the enhancement due to surgical damage, including ischemia and contusion or implantation of carmustine wafers. TTField-related characteristics included the use of TTFields, duration and compliance rate, and dermal complications. Progressive disease was defined based on the response assessment criteria for neuro-oncology for high-grade glioma.^17^ Data on the pattern of failure were also collected. Recurrences were classified as local when within 20 mm of the resection cavity and distant when more than 21 mm from the resection cavity or leptomeningeal dissemination.^18^ In addition, we collected information on reasons for not selecting or discontinuing TTFields.

### Statistical Analysis

Statistical analysis was performed using JMP Pro 16.0 (SAS Institute Japan) and R software (version 4.4.1; R Foundation). Linear regression analysis was used to examine the correlation between 2 continuous variables. To compare proportions between TTField users and nonusers, parametric data were compared using Pearson’s chi-square test. PFS was defined as the time between the date of adjuvant TMZ initiation and progression or last follow-up without recurrence, whereas OS was defined as the time between the date of adjuvant TMZ initiation and death or last follow-up. Multivariate logistic regression analysis was performed for the use of TTFields, with age, sex, caregiver, social status, JCS, paresis and aphasia before adjuvant TMZ, and extent of resection as covariates. Multivariate analysis using Cox proportional hazards analysis was planned for recurrence and survival with the use of TTFields as a covariate in addition to age, sex, KPS before adjuvant TMZ, extent of resection, and carmustine wafer implantation,^7,19^ as these were previously reported as prognostic factors in the EF-14 trial and other reports.^7,19^ Cox proportional hazards analysis was performed for patients for whom information on the promoter methylation status of *MGMT* was available or after multiple imputations via a chained equations model of missing data on the methylation status of *MGMT* as a sensitivity analysis. The pool of 50 imputed datasets was analyzed, and a pooled analysis was performed.

In addition, propensity score matching was performed to reduce selection bias caused by the difference in patients’ backgrounds between TTField users and nonusers.^20^ The model used to construct the propensity score included confounding factors such as age, sex, tumor location, extent of resection, KPS before adjuvant TMZ, JCS before adjuvant TMZ, and mutation status in *IDH* as predictors using TTFields.^21–24^ A 1:1 neighbor ratio matching was performed between the 2 groups, with a caliper set to 0.2. To compare the mean or prevalence of baseline covariates, we calculated the standardized difference between TTField users and nonusers.^25^ In addition, the distribution of patient’s age, a strong confounding factor between TTF use and prognosis, was compared with the variance ratio and 5 number summaries.^25^ After propensity score matching, the PFS and OS rates were compared using Kaplan–Meier analysis.

## Results

### Patient Background

**Figure 1.**
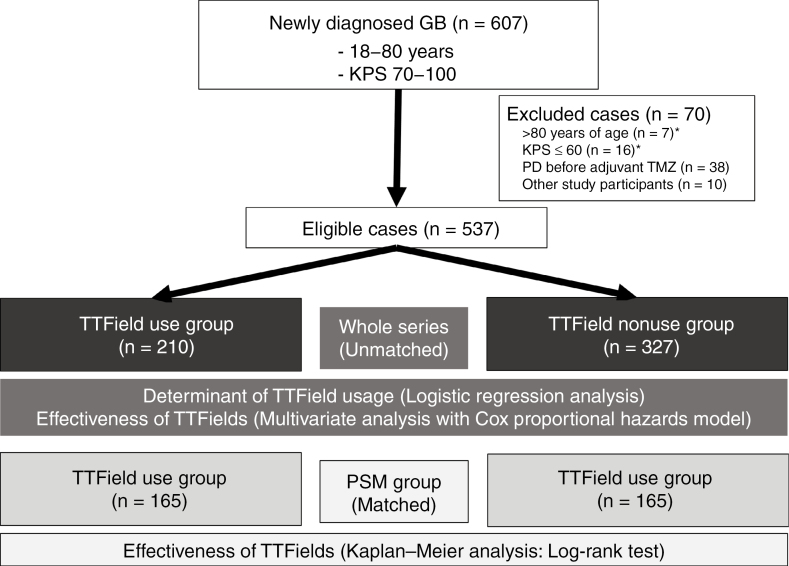
Patient flow in this retrospective study. GB, glioblastoma; KPS, Karnofsky Performance Status; *One patient was over 80 years old and had a KPS score of <60. A total of 537 patients in the whole series and 330 patients after propensity score matching (PSM) were analyzed. Logistic regression analysis was conducted to identify determinants of TTField usage, while multivariate analysis using the Cox proportional hazards model was performed to estimate the effectiveness of TTFields in the whole series. Kaplan–Meier analysis with the log-rank test was used to estimate the effectiveness of TTFields in the PSM group.

In total, 607 patients from 45 institutions were enrolled (Figure 1). However, 70 (12%) patients, including 7 over 80 years of age, 16 with a KPS score below 70 at the start of adjuvant TMZ, 38 with progressive disease, and 10 other study participants (KPS score of 60, 1 duplicate over 80 years of age) were excluded. Finally, 537 patients were analyzed in this study. Among them, 210 (39%) and 327 patients (61%) did and did not receive TTFields, respectively. The follow-up period was 1–55 months, with a median of 21 months.

### Determinant of TTField Usage

The reason for not choosing TTFields was determined from 297 of 327 patients (91%) using a multiple-response questionnaire. The reasons included patient refusal in 174 cases (59%), physician’s decision in 106 cases (36%), no caregiver in 36 cases (12%), and high cost in 6 cases (2%). Compared with chemotherapy and radiotherapy, the intention of the patient, family, and physician for or against TTField use was more strongly reflected in the determination of TTField use.

**Figure 2.**
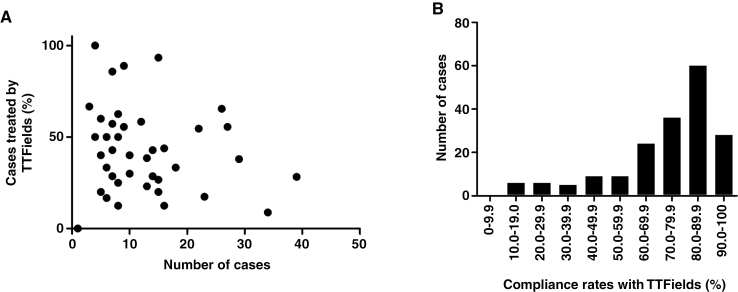
Tumor-treating field (TTField) usage rates according to institution and compliance rates with TTFields. (A) Scattered graph demonstrating the relationship between the total number of patients that met the inclusion criteria during the study period and patients who received TTField treatment at the 45 institutions. (B) Bar graph demonstrating the distribution of the average daily compliance rate with TTFields.

The proportion of TTField usage was compared according to multiple factors, such as institution, patient background, neurological findings, and treatments other than TTFields. TTField usage rates varied according to the institution. Moreover, no correlation was observed between the number of enrolled cases and the percentage of TTField use according to the institution (*R*^2^ = 0.01, *P* = .49) (Figure 2A). An imbalance in the distribution of age and categorical variables was observed between TTField users and nonusers (Table 1). Logistic regression analysis was performed to identify factors influencing TTField usage. Our analysis identified young age and spouse as a caregiver as significant factors for choosing TTFields. The odds ratio (95% confidence interval [CI]) for each significant factor was as follows. spouse versus others (2.1; 95% CI 1.3–3.4; *P* < .005), spouse versus living alone (8.0; 95% CI 18–35.5; *P* < .001), and age (0.97, 0.96–0.99; *P* < .0005).

**Table 1.** Characteristics of Patients According to TTField Use

		TTFields (*n* = 210) (%)	No TTFields (*n* = 327) (%)
Age	(years old) (mean + SD)	54.0 ± 12.3	60.0 ± 13.8
Sex	Male	132 (63)	185 (57)
	Female	78 (37)	142 (43)
Social status before disease onset	Employed	152 (73)	188 (58)
	Unemployed	57 (27)	137 (42)
Caregiver	Spouse	172 (82)	221 (68)
	Others	36 (17)	85 (26)
	Solitude	2 (1)	21 (6)
Tumor location	Frontal	81 (39)	110 (34)
	Temporal	59 (28)	103 (32)
	Parietal	50 (24)	80 (25)
	Occipital	5 (2)	12 (4)
	Insula	3 (1)	10 (3)
	Basal ganglia	4 (2)	4 (1)
	Thalamus	7 (3)	6 (2)
Extent of resection	No residual enhancing lesion	126 (60)	219 (67)
	Unmeasurable residual enhancement	39 (19)	42 (13)
	Measurable residual enhancement	31 (15)	42 (13)
	Biopsy	14 (7)	24 (7)
Implantation of carmustine wafers	Yes	64 (30)	97 (30)
	No	146 (70)	229 (70)
Photodynamic therapy	Yes	18 (9)	23 (7)
	No	192 (91)	304 (93)
Residual lesion before adjuvant TMZ^a^	No enhancing lesion	132 (63)	215 (66)
	Residual enhancement	78 (37)	111 (34)
KPS before adjuvant TMZ	100	36 (17)	40 (12)
	90	92 (44)	132 (40)
	80	40 (19)	73 (22)
	70	42 (20)	82 (25)
Japan Coma Scale before adjuvant TMZ	0	181 (86)	259 (80)
	1–3	29 (14)	66 (20)
Paresis before adjuvant TMZ	Absent	175 (84)	280 (86)
	Present	34 (16)	46 (14)
Apasia before adjuvant TMZ	Absent	190 (90)	280 (86)
	Present	20 (10)	45 (14)
Cycles of adjuvant TMZ	(Median cycles)	0–53 (12)	0–43 (12)
*MGMT*promoter methylation status	Methylated	44 (21)	69 (21)
	Unmethylated	62 (30)	66 (20)
	No data	104 (49)	192 (59)
Mutation status in the *IDH* gene	Wild-type	192 (92)	301 (95)
	Mutant	16 (8)	17(5)

Abbreviations: IDH, isocitrate dehydrogenase; KPS, Karnofsky Performance Status; *MGMT*, O6-methylguanine DNA methyltransferase; TMZ, temozolomide; TTFields, tumor-treating fields.

^a^One case was not examined due to asthma.

### Status After Starting TTFields

Information on the mean compliance rate during the period of use was collected for 187 patients (89%). The median compliance rate was 78.8%, with 113 (60%) and 92 patients (49%) achieving a compliance rate of >75% and >80%, respectively (Figure 2B). After examining the timing and reasons for the discontinuation of TTFields, we found that 185 of the 210 patients had discontinued TTField use at the time of the survey. Kaplan–Meier analysis revealed that the time from initiation to discontinuation ranged from 0 to 54 months (median 11 months). Among the patients that had discontinued TTField use, 61 (33%) did so within 1 month of the first recurrence. The reasons for discontinuing TTFields were determined in 173 (94%) patients. The most common reasons for discontinuation according to the duration of TTField usage were patients’ will (17 of 40 cases: 43%) at 1–3 months after TTField initiation, tumor progression (51 of 67 cases: 76%) at 4–12 months after initiation, and tumor progression (41 of 66 cases: 61%) at 13 months or later after initiation.

### Dermal Adverse Events in TTField Users

Skin disorders were present in 56% of the patients, among whom 48% had disorders requiring medical treatment and 8% had grade 3 or higher disorders based on Common Toxic Criteria for Adverse Events, version 4. Wound dehiscence was observed in a relatively high percentage of patients (6.7%).

### Effectiveness of TTFields

Univariate and multivariate analyses were performed to evaluate recurrence and survival in 537 patients who satisfied the EF-14 inclusion criteria. TTField users were more likely younger, had a job before disease onset, and had a spouse than nonusers (Table 1). The proportion of patients with a JCS of 0 before adjuvant treatment, which indicates alert consciousness, also tended to be higher among TTField users than among nonusers (Table 1).

During the study period, 166 (79.1%) TTField users and 251 (76.8%) nonusers had a recurrence. Supplementary Table 1 summarizes the pattern of recurrence in these TTField users and nonusers. Although the Pearson’s chi-square test showed no significant difference in the pattern of recurrence between TTField users and nonusers, a trend toward fewer local recurrences and more distant recurrences was observed among TTField users.

**Figure 3.**
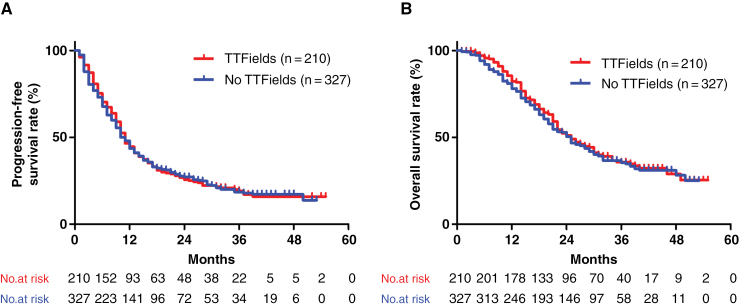
Progression-free survival (A) and overall survival (B) in all patients who received radiation and temozolomide therapy with or without tumor-treating fields (TTFields). The median PFS was 11 and 10 months in TTField users and nonusers, respectively. The median OS was 25 months in both groups. There were no significant differences in PFS and OS between TTField users and nonusers (*P* = .83 and .57; log-rank test).

The median PFS was 11 and 10 months in TTField users and nonusers, respectively. When defining “TTFields beyond progression” as the date of discontinuation >2 months after the date of recurrence, 53 (32.7%) of 162 patients with recurrence continued to use TTFields beyond progression. During the study period, 126 (60.0%) TTField users and 192 (58.7%) TTField nonusers died. The median OS was 25 months in both groups, whereas the 2-year OS rate was 51.7% and 50.3% in TTField users and nonusers, respectively. No significant differences in PFS and OS were observed between TTField users and nonusers (*P* = .83 and .57; log-rank test) (Figure 3A and B). Univariate analysis was also performed for other factors. Notably, prognostic factors for PFS included sex, caregiver, tumor location, JCS, aphasia before adjuvant TMZ, *MGMT* promoter methylation status, and *IDH* gene mutation status (Supplementary Table 2). Prognostic factors for OS included age, sex, residual lesion, KPS, consciousness level before adjuvant TMZ, *MGMT* gene promoter methylation status, and *IDH* gene mutation status (Supplementary Table 2).

Multivariate analysis using the Cox proportional hazards model revealed that age and sex were prognostic factors for PFS, whereas age, sex, and KPS before adjuvant TMZ were prognostic factors for OS. However, TTField use was not a prognostic factor for PFS or OS (Table 2). In addition, we performed a multivariate analysis using *MGMT* methylation status for 241 cases, including 135 TTField users and 106 nonusers, for whom data on the methylation status of the *MGMT* promoter were available (Supplementary Table 3). The MGMT methylation status was a prognostic factor for recurrence and survival, whereas TTField use was not. The pooled analysis of 50 datasets with multiple imputation of data on *MGMT* gene promoter methylation status revealed that TTField use was not a prognostic factor for PFS (hazard ratio: 0.90, 95% CI 0.74–1.11; *P* = .32) or OS (hazard ratio: 0.95, 95% CI 0.75–1.20; *P* = .65).

**Table 2.** Multivariate Analysis of the Progression-Free and Overall Survival Rates in This Study

		Progression-Free Survival Rate		Overall Survival Rate	
		Adjusted HR (95% CI)	*P* Value	Adjusted HR (95% CI)	*P* Value
Age	Age	1.01 (1.00–1.01)	**.014**	1.01 (1.00–1.02)	**.006**
Sex	Female (reference)	1		1	
	Male	1.31 (1.08–1.61)	**.0075**	1.43 (1.14–1.81)	**.002**
Extent of resection	Biopsy (reference)	1		1	
	Measurable residual enhancement	1.00 (0.64–1.57)	.31	0.83 (0.51–1.37)	.47
	Unmeasurable residual enhancement	0.85 (0.54–1.35)	.15	0.73 (0.44–1.21)	.22
	No residual enhancing lesion	0.93 (0.65–1.45)	.89	0.71 (0.46–1.09)	.12
Implantation of carmustine wafers	No	1		1	
	Yes	1.10 (0.88–1.36)	.41	1.13 (0.88–1.45)	.33
KPS before adjuvant TMZ	70 (reference)	1		1	
	80	0.92 (0.68–1.23)	.58	0.76 (0.55–1.05)	.097
	90	0.81 (0.63–1.05)	.11	0.65 (0.49–0.87)	**.004**
	100	0.77 (0.55–1.10)	.15	0.54 (0.36–0.81)	**.003**
TTFields use	No use (reference)	1		1	
	Use	0.98 (0.80–1.20)	.81	0.99 (0.78–1.25)	.93

Values in bold indicate statistical significance at *P* < .05.

Abbreviations: CI, confidence interval; HR = Hazard ratio; KPS, Karnofsky Performance Status; TMZ, temozolomide; TTFields, tumor-treating fields.

After propensity score matching, we confirmed the balance between TTField users (*n* = 165) and nonusers (*n* = 165) according to the standardized difference (Figure 1, Supplementary Table 4). The standardized difference was <0.1 for all covariates, except the prevalence of patients with basal ganglia glioblastoma. In addition, we confirmed that patients’ age was balanced based on the variance ratio (0.92: variance in TTField users to that in TTField nonusers) and 5 number summaries of patients’ age (Supplementary Table 5). These results suggested that the model based on propensity score matching had a balanced background between TTField users and nonusers. Consistent with the results of the univariate and multivariate analyses of all cases, no differences in PFS and OS were observed between TTField users and nonusers in propensity score matching (Supplementary Figure 1).

We further analyzed the relationship between survival and the compliance rate. No improvements in PFS or OS were found at cutoff values of 75% (Supplementary Figure 2), 80%, 85%, and 90% (data not shown).

## Discussion

This study provides data on TTField usage rates in patients satisfying the EF-14 trial inclusion criteria. This study also identifies determinants of TTField use; duration, compliance rates, and discontinuation after TTField introduction; safety; and effects of TTField on recurrence and survival based on data obtained from 45 institutions 2 years after the approval of TTFields in Japan. Notably, our analysis identified age and caregiver status as independent determinants of TTField use. A large proportion of patients discontinued treatment of their own volition, especially within 3 months of TTField initiation. Our univariate and multivariate analyses and comparison of 2 groups after propensity score matching revealed no difference in PFS and OS between TTField users and nonusers.

Unlike in clinical trials, decisions regarding TTField use in actual clinical practice are made by the patients themselves, their caregivers, and medical doctors after considering the burden of continuing TTField therapy, including the need for shaving, 24-hour electrode application, assistance with TTField device application, skin complications, and expected real-world benefits on tumor control. In the present study, only 39% of the patients who satisfied the EF-14 inclusion criteria accepted TTField therapy. This percentage was comparable to the 36% obtained from real-world data for single centers in the United States and Germany.^26,27^ A subgroup analysis of the EF-14 trial revealed that elderly patients are expected to benefit more from TTFields than younger patients.^7^ However, a single-center study in the United States and Germany reported that elderly patients tend to avoid the use of TTFields in the real world.^21–23^ Consistent with their data, our findings showed that aging was a negative independent factor that influenced TTField use. In addition, this study revealed that the type of caregiver was an independent determinant of TTField usage. These results suggest that patient’s living environment and characteristics strongly influence their acceptance of TTFields in the real world. Moreover, since EF-14 was an open-label trial without a sham device, the results could have been influenced by patient adherence, selection, or other biases.^8^ Moreover, these study design characteristics could influence the acceptance of TTFields as a standard of care provided by physicians for newly diagnosed GB. Differences in the interpretation of EF-14 may have contributed to large differences in the rate of its introduction among institutions, as shown in Figure 2A.

One of the characteristics of this treatment is the high rate of discontinuation at an early stage due to patient refusal despite the absence of tumor progression. Some patients may perceive more psychological and emotional burdens than expected and discontinue TTFields early after initiation. However, the median treatment duration was 11 months, which was longer than that in the EF-14 trial (8.2 months)^7^ and other studies (7.2–9 months).^27,28^ Only 17 of 173 patients with available information had discontinued early based on the patient’s will, and tumor progression was the main cause of TTF discontinuation after 3 months. Therefore, the difference in the treatment duration of TTFields may reflect the duration of PFS (11 and 6.7 months in the present study and EF-14 trial, respectively).

Regarding adverse events, a high rate of skin problems was expected due to the hot and humid climate in Japan and the longer treatment period; however, only 56% of our patients developed skin symptoms, which was similar to the 54% in the EF-14 trial.^7^

The effect of TTFields on recurrence patterns, PFS, and OS was analyzed. Regarding recurrence pattern, the rate of local recurrence was lower in TTField users than in nonusers, consistent with a previous report.^18^ This finding suggests that TTFields contribute to local tumor control. TTField users had a median survival of 25.0 months and a 2-year survival rate of 51.7%. This survival rate was comparable to the 2-year survival rate of 53.6% in a Japanese post-marketing study of 40 newly diagnosed cases of GB, in which no difference in age (median age: 59 years), KPS (65% with a score of 90–100), and resection rate (gross total resection of enhanced lesion: 57.5%) was observed.^29^ However, univariate and multivariate analyses showed no difference in PFS or OS between TTField users and nonusers. This could be attributed to several possible reasons. The first is the underestimation of the effect of TTFields due to low compliance rates. Indeed, only 49% of the patients included in this study achieved at least 80% compliance. This compliance rate was comparable to the 46% in the EF-14 trial,^30^ suggesting that a low compliance rate was an unlikely reason for the lack of efficacy. The second reason is the excellent survival rate of TTField nonusers in this study (25 months) compared with that of TTField nonusers in the EF-14 trial (16.0 months). To clarify the reason for this difference, we compared the backgrounds of TTField nonusers in our series and those in the EF-14 trial. Accordingly, more TTField nonusers in the present study than those in the EF-14 trial had total resections of contrast-enhancing lesions (67% vs. 54%, respectively) and *MGMT* gene promoter methylation (52% vs. 36%, respectively); however, fewer TTField nonusers in the present study than those in the EF-14 trial had KPS scores of 90–100 (52% vs. 65%, respectively) and age of < 65 years (54% vs. 80%, respectively).^7^ Based on these findings, the patient background in the present study is not biased toward a favorable prognosis. Alternatively, multiple reports have suggested biological differences between Caucasian and Asian GBs.^10,31^ Therefore, we compared the survival time in this study to that in a prospective study conducted in Japan from 2010 to 2012^9^ and in a retrospective study based on a large number of Korean patients using real-world data.^32^ The median OS time following the Stupp regimen was 20.3 months in the JCOG0911 study, which explored the additive effect of interferon-beta to radiation and temozolomide on newly diagnosed glioblastoma.^9^ The median patient age was comparable between the RT/TMZ group in the JCOG0911 study and TTField nonusers in our study (61 vs. 64 years). However, the percentage of patients with total resection of enhanced lesion was lower in the JCOG0911 study than in our study (49% vs. 67%; Table 1). Roh et al. reported a median OS time of 20.8 months among patients treated from 2005 to 2013 at Yonsei University.^32^ Although the median age was 57 years in the previous study,^32^ the preoperative and postoperative median KPS was 70, and 66% of patients had an unmethylated promoter in *MGMT*. Since our study excluded patients with a KPS < 70 and only 48% of our patients had an unmethylated *MGMT* promoter, a longer OS time is expected in our patients than in those in Roh et al.’s study. In addition, Kim et al. reported the OS time based on the response following concomitant radiation and temozolomide and 6 cycles of adjuvant temozolomide.^33^ The OS times were 8.0 and 25.0–27.0 months in patients with and without progression during the initial and adjuvant phases of treatment, respectively. Although there was a difference between the end of the concomitant and adjuvant treatment phases, the exclusion of patients with progression during the initial treatment phase from the analysis could have significantly impacted the survival time. Based on these reports and considering the backgrounds of the patients in this study, the median survival time of 25 months for RT/TMZ was considered valid in this report. We considered the possibility that such racial differences may have influenced survival differences in the control group and that this population may benefit less from TTFields. Third, certain clinical and molecular characteristics may predispose patients to benefit more from TTFields. A subgroup analysis of the EF-14 trial showed that patients who underwent incomplete resection were elderly, and those with poor KPS tended to have decreased hazard ratios and increased benefits from TTFields.^7^ This study found a trend toward complete resection of enhanced lesions, elderly patients, and poor KPS, where the latter 2 factors potentially increased the benefits from TTFields. On the contrary, considering that TTField therapy targets local control,^34^ it may not have been effective in patients with more complete resection and a lower local failure rate.^35^ Regarding molecular predictive factors, comprehensive genomic profiling revealed predictive biomarkers associated with response to TTFields.^36^ Driver alterations in the Neurofibromatosis type 1 (*NF1*) gene and wild-type Phosphatidylinositol-4,5-Bisphosphate 3-Kinase Catalytic Subunit Alpha (*PIK3CA*) gene and the absence of aberrations in *EGFR*, such as amplification and mutations, including the type III variant or fusion, were predictive for the effect of TTFields. Although the mutation frequency in *NF1* and *PIC3CA* was comparable, the low frequency of the amplification or alteration of *EGFR* is a characteristic of Asian patients with GB.^10,31^ Unfortunately, these differences in molecular profile do not explain the underlying mechanism for the ineffectiveness of TTFields in this study. A more comprehensive molecular analysis of Japanese patients with GB may help answer this question.

This study had some limitations. First, the EF-14 trial randomly stratified their patients based on *MGMT* promoter methylation status; however, insufficient data on the promoter methylation status of *MGMT* is a limitation of this study. The promoter methylation status of *MGMT* was obtained only for 106 patients (50.5%) in the TTField user group and 135 patients (41%) in the TTField nonuser group, and the methylation analysis method may differ between institutions. Consequently, the proportion of patients with *MGMT* promoter methylation may be biased between the 2 groups. However, multivariate analysis in patients with information on the promoter methylation status and in all patients using multiple imputations of missing data on the promoter methylation status did not demonstrate the effect of TTField on PFS or OS. Second, given that we did not collect information on salvage treatment, the effect of TTFields may have been masked by differences in salvage treatment. However, the median PFS was comparable between the 2 groups. In standard clinical practice, the treatment modalities for progressive GB are limited; therefore, the effect of salvage treatment on OS time was limited. Third, although we included most patients who met the inclusion criteria during the study period, this retrospective study could not eliminate biases such as variations in imaging timing, inconsistencies in treatment schedules, and patient preferences influencing treatment selection.

## Conclusions

This multi-institutional study retrospectively reviewed and presented data on TTField use among patients who satisfied the EF-14 trial inclusion criteria 2 years after the approval of TTFields in Japan. Notably, we found that only 39% of patients opted to receive TTFields after radiation and TMZ therapy. Moreover, our findings identified age and caregiver as independent determinants of TTField use. A significant proportion of patients discontinued their treatment of their own volition, especially within 3 months after TTField initiation. Despite the comparable compliance rates between our study and the EF-14 trial, substantial effects on PFS and OS were not observed in univariate and multivariate analyses and in comparison between TTField users and nonusers after propensity score matching.

## Supplementary Material

vdae176_suppl_Supplementary_Figures

vdae176_suppl_Supplementary_Tables

## References

[1] Stupp R, Mason WP, van den Bent MJ, et al; European Organisation for Research and Treatment of Cancer Brain Tumor and Radiotherapy Groups. Radiotherapy plus concomitant and adjuvant temozolomide for glioblastoma. N Engl J Med. 2005;352(10):987–996. 15758009 10.1056/NEJMoa043330

[2] Pallud J, Audureau E, Noel G, et al Long-term results of carmustine wafer implantation for newly diagnosed glioblastomas: a controlled propensity-matched analysis of a French multicenter cohort. Neuro Oncol. 2015;12(12):201517. 10.1093/neuonc/nov126 PMC4633930 26185110

[3] Aoki T, Nishikawa R, Sugiyama K, et al; NPC-08 study group. NPC-08 study group. A multicenter phase I/II study of the BCNU implant (Gliadel(®) Wafer. Neurol Med Chir (Tokyo). 2014;54(4):290–301. 24739422 10.2176/nmc.oa2013-0112 PMC4533485

[4] Westphal M, Hilt DC, Bortey E, et al A phase 3 trial of local chemotherapy with biodegradable carmustine (BCNU) wafers (Gliadel Wafers) in patients with primary malignant glioma. Neuro Oncol. 2003;5(2):79–88. 12672279 10.1215/S1522-8517-02-00023-6 PMC1920672

[5] Muragaki Y, Akimoto J, Maruyama T, et al Phase II clinical study on intraoperative photodynamic therapy with talaporfin sodium and semiconductor laser in patients with malignant brain tumors. J Neurosurg. 2013;119(4):845–852. 23952800 10.3171/2013.7.JNS13415

[6] Stupp R, Wong ET, Kanner AA, et al NovoTTF-100A versus physician’s choice chemotherapy in recurrent glioblastoma: a randomised phase III trial of a novel treatment modality. Eur J Cancer. 2012;48(14):2192–2202. 22608262 10.1016/j.ejca.2012.04.011

[7] Stupp R, Taillibert S, Kanner A, et al Effect of tumor-treating fields plus maintenance temozolomide vs maintenance temozolomide alone on survival in patients with glioblastoma: a randomized clinical trial. JAMA. 2017;318(23):2306–2316. 29260225 10.1001/jama.2017.18718 PMC5820703

[8] Lassman AB, Joanta-Gomez AE, Pan PC, Wick W. Current usage of tumor treating fields for glioblastoma. Neurooncol Adv. 2020;2(1):vdaa069. 32666048 10.1093/noajnl/vdaa069 PMC7345837

[9] Wakabayashi T, Natsume A, Mizusawa J, et al; Members of Japan Clinical Oncology Group Brain Tumor Study Group (JCOG-BTSG). JCOG0911 Integra study: a randomized screening phase II trial of interferon β plus temozolomide in comparison with temozolomide alone for newly diagnosed glioblastoma. J Neurooncol. 2018;138(3):627–636. 29557060 10.1007/s11060-018-2831-7 PMC5999164

[10] Lassman AB, Aldape KD, Ansell PJ, et al Epidermal growth factor receptor (EGFR) amplification rates observed in screening patients for randomized trials in glioblastoma. J Neurooncol. 2019;144(1):205–210. 31273577 10.1007/s11060-019-03222-y PMC8082754

[11] Bohn A, Braley A, Rodriguez de la Vega P, Zevallos JC, Barengo NC. The association between race and survival in glioblastoma patients in the US: a retrospective cohort study. PLoS One. 2018;13(6):e0198581. 29927955 10.1371/journal.pone.0198581 PMC6013158

[12] Kim CY, Paek SH, Nam DH, et al Tumor treating fields plus temozolomide for newly diagnosed glioblastoma: a sub-group analysis of Korean patients in the EF-14 phase 3 trial. J Neurooncol. 2020;146(3):399–406. 32020470 10.1007/s11060-019-03361-2

[13] She L, Gong X, Su L, Liu C. Effectiveness and safety of tumor-treating fields therapy for glioblastoma: a single-center study in a Chinese cohort. Front Neurol. 2022;13:1042888. 36698900 10.3389/fneur.2022.1042888 PMC9869119

[14] Louis DN, Ohgaki H, Wiestler OD, et al WHO Classification and Grading of Tumours of the Central Nervous System. Lyon: IARC Press, International Agency for Research on Cancer; 2016.

[15] Macdonald DR, Cascino TL, Schold SC Jr, Cairncross JG. Response criteria for phase II studies of supratentorial malignant glioma. J Clin Oncol. 1990;8(7):1277–1280. 2358840 10.1200/JCO.1990.8.7.1277

[16] Ohta T, Nakatomi H, Matsutani M. Classification of disturbance of consciousness. In: Ohta T, Matsutani M, Nozaki K, eds. Neurosurgery. Version 13. Kyoto: Kinpodo; 2021:224–228.

[17] Wen PY, Macdonald DR, Reardon DA, et al Updated response assessment criteria for high-grade gliomas: response assessment in neuro-oncology working group. J Clin Oncol. 2010;28(11):1963–1972. 20231676 10.1200/JCO.2009.26.3541

[18] Ali AS, Lombardo J, Niazi MZ, et al Concurrent chemoradiation and Tumor Treating Fields (TTFields, 200 kHz) for patients with newly diagnosed glioblastoma: patterns of progression in a single institution pilot study. J Neurooncol. 2022;160(2):345–350. 36355259 10.1007/s11060-022-04146-w

[19] Roux A, Peeters S, Zanello M, et al Extent of resection and carmustine wafer implantation safely improve survival in patients with a newly diagnosed glioblastoma: a single center experience of current practice. J Neurooncol. 2017;135(1):83–92. 28669011 10.1007/s11060-017-2551-4

[20] Austin PC. An introduction to propensity score methods for reducing the effects of confounding in observational studies. Multivariate Behav Res. 2011;46(3):399–424. 21818162 10.1080/00273171.2011.568786 PMC3144483

[21] Blakstad H, Brekke J, Rahman MA, et al Survival in a consecutive series of 467 glioblastoma patients: association with prognostic factors and treatment at recurrence at two independent institutions. PLoS One. 2023;18(2):e0281166. 36730349 10.1371/journal.pone.0281166 PMC9894455

[22] Fyllingen EH, Bø LE, Reinertsen I, et al Survival of glioblastoma in relation to tumor location: a statistical tumor atlas of a population-based cohort. Acta Neurochir (Wien). 2021;163(7):1895–1905. 33742279 10.1007/s00701-021-04802-6 PMC8195961

[23] Patil N, Somasundaram E, Waite KA, et al Independently validated sex-specific nomograms for predicting survival in patients with newly diagnosed glioblastoma: NRG Oncology RTOG 0525 and 0825. J Neurooncol. 2021;155(3):363–372. 34761331 10.1007/s11060-021-03886-5 PMC8651582

[24] Li J, Wang M, Won M, et al Validation and simplification of the Radiation Therapy Oncology Group recursive partitioning analysis classification for glioblastoma. Int J Radiat Oncol Biol Phys. 2011;81(3):623–630. 20888136 10.1016/j.ijrobp.2010.06.012 PMC3783211

[25] Austin PC. Balance diagnostics for comparing the distribution of baseline covariates between treatment groups in propensity-score matched samples. Stat Med. 2009;28(25):3083–3107. 19757444 10.1002/sim.3697 PMC3472075

[26] Liu Y, Strawderman MS, Warren KT, et al Clinical efficacy of tumor treating fields for newly diagnosed glioblastoma. Anticancer Res. 2020;40(10):5801–5806. 32988908 10.21873/anticanres.14597

[27] Onken J, Staub-Bartelt F, Vajkoczy P, Misch M. Acceptance and compliance of TTFields treatment among high grade glioma patients. J Neurooncol. 2018;139(1):177–184. 29644485 10.1007/s11060-018-2858-9

[28] Ballo MT, Qualls KW, Michael LM, et al Determinants of tumor treating field usage in patients with primary glioblastoma: a single institutional experience. Neurooncol. Adv.. 2022;4(1):vdac150. 36249289 10.1093/noajnl/vdac150 PMC9555297

[29] Nishikawa R, Yamasaki F, Arakawa Y, et al Safety and efficacy of tumour-treating fields (TTFields) therapy for newly diagnosed glioblastoma in Japanese patients using the Novo-TTF System: a prospective post-approval study. Jpn J Clin Oncol. 2023;53(5):371–377. 36647599 10.1093/jjco/hyad001 PMC10150168

[30] Toms SA, Kim CY, Nicholas G, Ram Z. Increased compliance with tumor treating fields therapy is prognostic for improved survival in the treatment of glioblastoma: a subgroup analysis of the EF-14 phase III trial. J Neurooncol. 2019;141(2):467–473. 30506499 10.1007/s11060-018-03057-z PMC6342854

[31] Koo H, Choi SW, Cho HJ, et al Ethnic delineation of primary glioblastoma genome. Cancer Med. 2020;9(19):7352–7359. 32794373 10.1002/cam4.3370 PMC7541127

[32] Roh TH, Park HH, Kang SG, et al Long-term outcomes of concomitant chemoradiotherapy with temozolomide for newly diagnosed glioblastoma patients: a single-center analysis. Medicine (Baltimore). 2017;96(27):e7422. 28682902 10.1097/MD.0000000000007422 PMC5502175

[33] Kim SK, Kim TM, Lee ST, et al The survival significance of a measurable enhancing lesion after completing standard treatment for newly diagnosed glioblastoma. J Clin Neurosci. 2016;34:145–150. 27475318 10.1016/j.jocn.2016.06.014

[34] Wenger C, Salvador R, Basser PJ, Miranda PC. Improving tumor treating fields treatment efficacy in patients with glioblastoma using personalized array layouts. Int J Radiat Oncol Biol Phys. 2016;94(5):1137–1143. 26883559 10.1016/j.ijrobp.2015.11.042

[35] Yoo J, Yoon SJ, Kim KH, et al Patterns of recurrence according to the extent of resection in patients with IDH-wild-type glioblastoma: a retrospective study. J Neurosurg. 2021;137(2):533–543. 34972087 10.3171/2021.10.JNS211491

[36] Pandey M, Xiu J, Mittal S, et al Molecular alterations associated with improved outcome in patients with glioblastoma treated with Tumor-Treating Fields. Neurooncol Adv. 2022;4(1):vdac096. 35821680 10.1093/noajnl/vdac096 PMC9270729

