# Supplementary material for: Impact of tumor-treating fields on the survival of Japanese patients with newly diagnosed glioblastoma: A multicenter, retrospective cohort study
Source: Neurooncol Adv. 2024 Nov 28;6(1):vdae176. doi: 10.1093/noajnl/vdae176 (PMC11629686; doi:10.1093/noajnl/vdae176)
Supplement: vdae176_suppl_Supplementary_Figures [file vdae176_suppl_Supplementary_Figures.docx]

**
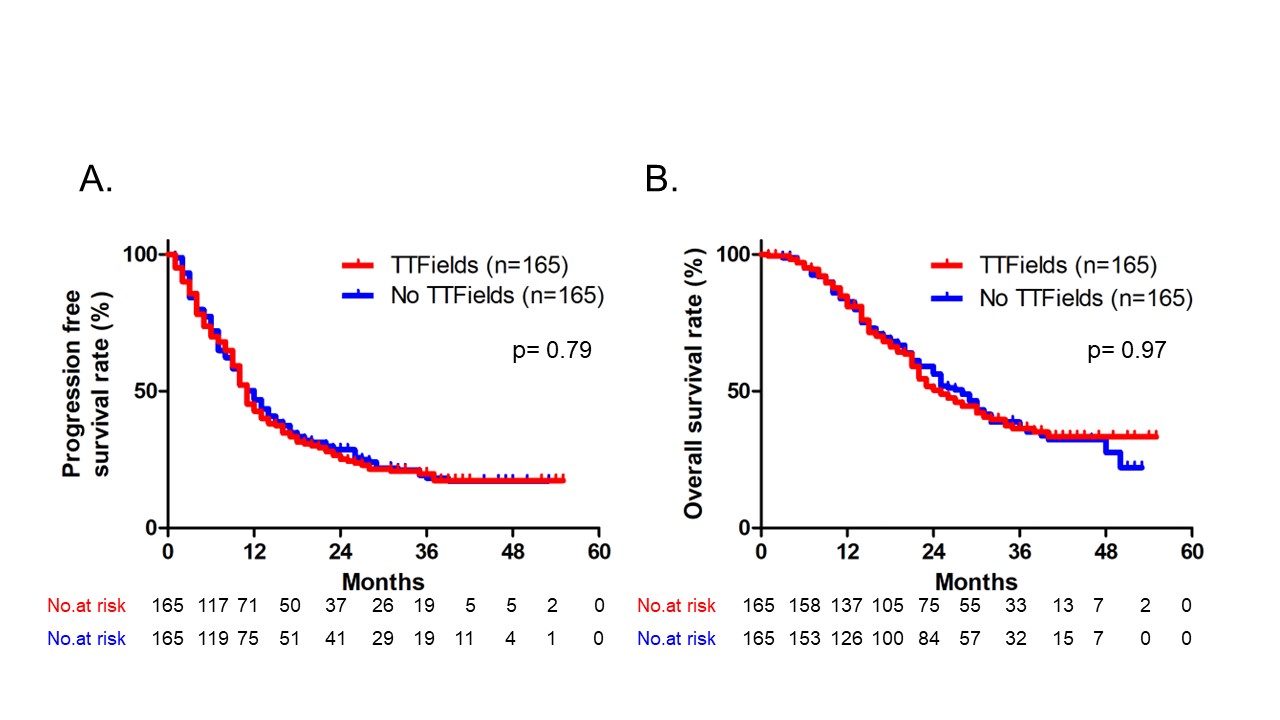
Supplemental Fig. 1.** Progression-free survival (A) and overall survival (OS) (B) in selected patients with propensity score matching who received radiation and temozolomide therapy with or without TTFields. The median PFS was 11 and 12 months in TTField users and nonuser, respectively. The median OS was 25 and 28 months in TTField users and nonusers, respectively. No significant differences in PFS and OS were observed between TTField users and nonusers (p = 0.79 and 0.97; log-rank test)


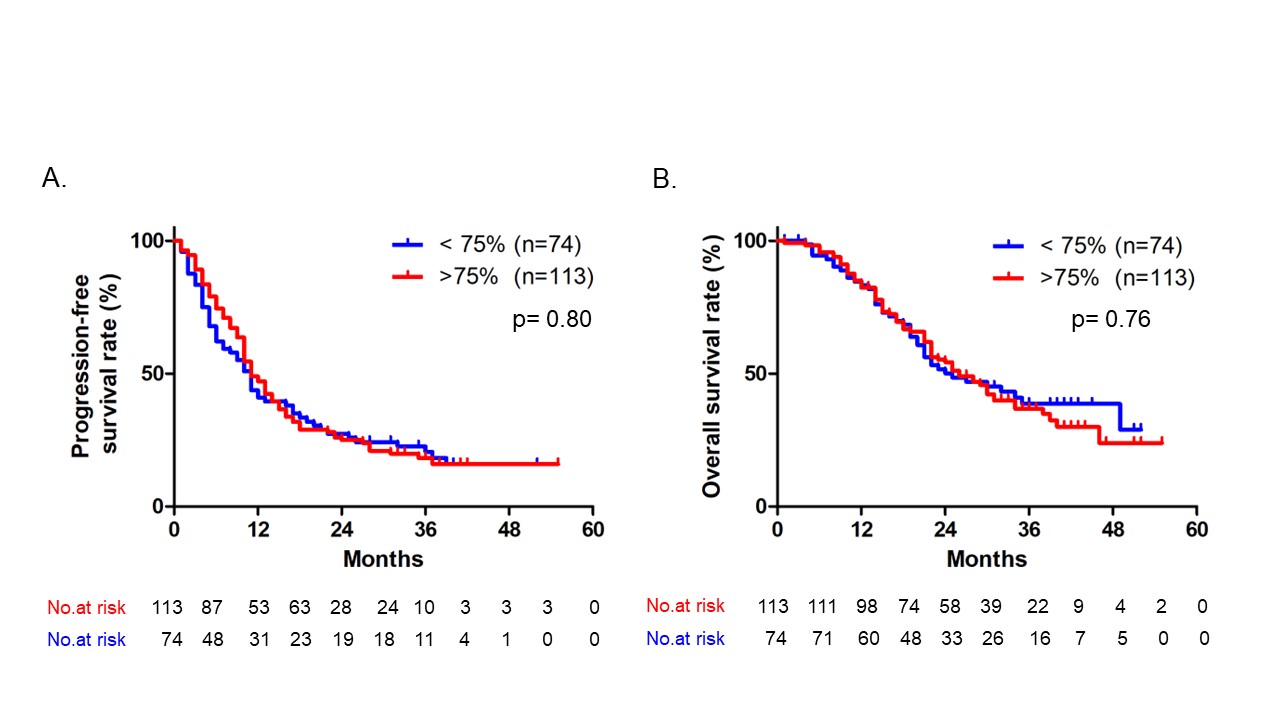
**Supplemental Fig. 2.** Progression-free survival (A) and overall survival (OS) (B) in the high (>75%) and low (<75%) compliance rate groups. The median PFS was 11 months in TTFields users and nonusers. The median OS was 26 months and 25 months in the high and low compliance groups, respectively. No significant differences in PFS and OS were observed between TTFields users and nonusers (p = 0.80 and 0.76; log-rank test)
