# Supplementary material for: Impact of tumor-treating fields on the survival of Japanese patients with newly diagnosed glioblastoma: A multicenter, retrospective cohort study
Source: Neurooncol Adv. 2024 Nov 28;6(1):vdae176. doi: 10.1093/noajnl/vdae176 (PMC11629686; doi:10.1093/noajnl/vdae176)
Supplement: Supplementary file 2 [file vdae176_suppl_Supplementary_Tables.docx]

Supplemental Table 1. The pattern of recurrence according to TTField use

Site of recurrence	With TTFields (n = 166)	Without TTFields (n = 251)	p value
Local (%)	89 (55)	155 (62)	0.30[a]
Local + distant (%)	19 (12)	23 (9)	
Distant (%)	54 (33)	64 (25)	
Unknown (%)	4 (2)	9 (4)	

Abbereviations: TTFields= tumor-treating fields; a= Pearson chi-square test

Supplemental Table 2 . Univariate analysis of progression–free survival and overal survival rates in this study

		Number of cases	Progression-free survival (95% CI)	p value[b]	Overal survival (95% CI)	p value[b]
Age	<60 years old	261	11 (10–13)	0.2067	28 (23–32)	**0.029**
	≥60 years old	276	10 (9–12)		23 (21–25)	
Sex	Male	317	10 (8–11)	**0.0088**	22 (20–25)	**0.0054**
	Female	220	13 (10–15)		28 (25–36)	
Social status	Employed	339	11 (10–12)	0.643	25 (23–29)	0.2716
	Unemployed	194	10 (9–13)		22 (20–27)	
Caregiver	Spouse	393	10 (9–11)	**0.0439**	24 (22–28)	0.3200
	Others	121	11 (9–14)		24 (19–27)	
	Solitude	23	28 (15–35)		38 (25–)	
Tumor location	Frontal	191	12 (10–15)	**0.0132**	25 (23–31)	0.2489
	Temporal	161	9 (6–10)		21 (19–23)	
	Parietal	130	11 (9–14)		29 (23–36)	
	Occipital	17	12 (3–17)		25 (11–)	
	Insula	13	23 (9–)		34 (19–)	
	Basal ganglia	8	13 (5–19)		30 (11–)	
	Thalamus	13	10 (7–18)		20 (15–32)	
Extent of resection	No residual enhanced lesion	345	10 (9–11)	0.6383	26 (23–30)	0.0968
	Residual unmeserable enhancement	81	14 (10–17)		25 (21–30)	
	Residual meserable enhancement	72	9 (8–12)		22 (17–28)	
	Biopsy	38	11 (8–17)		18 (12–24)	
Implantation of carmustine wafers	Yes	161	10 (7–12)	0.3983	24 (21–28)	0.4319
	No	375	11 (10–12)		25 (22–29)	
Photodynamic therapy	Yes	41	13 (6–29)	0.3913	24 (13–48)	0.859
	No	496	10 (10–12)		25 (22–27)	
Residual lesion before adjuvant TMZ	No enhanced lesion	347	11 (10–12)	0.1102	28 (24–31)	**0.0116**
	Residual enhancement	188	10 (9–12)		22 (19–24)	
KPS before adjuvant TMZ	100	76	12 (10–15)	0.0764	35 (25–)	**0.0003**
	90	224	11 (10–13)		26 (22–30)	
	80	113	10 (8–13)		25 (21–29)	
	70	124	9 (7–10)		21 (17–23)	
Japan Coma Scale before adjuvant TMZ	0	439	11 (10–13)	**0.0004**	26 (24–30)	**<0.0001**
	1–3	95	7 (5–10)		17 (12–21)	
Paresis before adjuvant TMZ	Absent	455	10 (10–12)	0.8496	25 (22–27)	0.9015
	Present	80	11 (9–16)		25 (22–32)	
Aphasia before adjuvant TMZ	Absent	469	11 (10–12)	**0.0177**	25 (23–29)	0.0592
	Present	65	8 (6–10)		22 (17–28)	
MGMT promoter methylation status	Methylated	128	13 (10–17)	**0.0081**	34 (24–28)	**0.0002**
	Unmethylated	113	9 (7–10)		22 (17–24)	
Mutation status in the *IDH* gene	Wild–type	492	10 (9–11)	**0.0003**	24 (22–26)	**0.0101**
	Mutant	33	31 (10–)		NR (21–)	
Institutes	>39.1%[a]	230	11 (10–13)	0.6326	24 (21–28)	0.7058
	≤ 39.1%[a]	306	10 (9–12)		25 (22–29)	
TTFields use	No	328	10 (9–12)	0.836	25 (21–28)	0.57
	Yes	209	11 (10–12)		25 (22–30)	

Abbereviations: TMZ, temozolomide; KPS, Karnofsky Performance Status; MGMT, O6-methylguanine DNA methyltransferase; IDH, isocitrate dehydrogenase; TTFields, tumor-treating fields; CI, confidence interval; NR, not reached; a, the cutoff value was the median TTField use value in each institute; b, log-rank test

Supplemental Table 3. Multivariate analysis of the progression–free and overall survival rates in the patients with the information on promoter methylation status in *MGMT* gene

		Progression–free survival rate		Overall survival rate	
		Adjusted HR (95% CI)	p–value	Adjusted HR (95% CI)	p–value
Age	Age	1.00 (0.99–1.02)	0.24	1.01 (0.99–1.03)	0.055
Sex	Female (reference)	1		1	
	Male	1.19 (0.87–1.62)	0.27	1.36 (0.95–1.95)	0.094
Extent of resection	Biopsy (reference)	1		1	
	Measurable residual enhancement	0.80 (0.39–1.66)	0.55	0.62 (0.28–1.36)	0.229
	Unmeasurable residual enhancement	0.75 (0.37–1.52)	0.42	0.48 (0.22–1.03)	0.058
	No residual enhancing lesion	0.81 (0.43–1.53)	0.52	0.45 (0.28–0.90)	**0.023**
Implantation of carmustine wafers	No	1		1	
	Yes	1.22 (0.88–1.70)	0.24	1.52 (1.05-2.21)	**0.027**
KPS before adjuvant TMZ	70 (reference)	1		1	
	80	0.87 (0.56–1.34)	0.52	0.90 (0.55–1.44)	0.890
	90	0.70 (0.48–1.02)	0.061	0.66 (0.43–1.01)	0.056
	100	0.80 (0.46–1.38)	0.42	0.73 (0.39–1.39)	0.34
Promoter methylation status in the *MGMT* gene	Methylated	1		1	
	Unmethylated	1.48 (1.09-1.99)	**0.011**	1.89 (1.33-2.68)	**0.0004**
TTFields use	No use (reference)	1		1	
	Use	1.03 (0.76–1.39)	0.85	1.09 (0.77–1.52)	0.63

Abbereviations: TMZ, temozolomide; KPS, Karnofsky Performance Status; TTFields, tumor-treating fields; MGMT, O6-methylguanine DNA methyltransferase; IDH, isocitrate dehydrogenase; HR. hazard ratio; CI, confidence interval

Supplemental Table 4. Characteristics of patients according to TTField use in the propensity score matched sample

		TTFields (n=165)	No TTFields (n=165)	Standarized difference
Age (mean, standard deviation)		55.2 ± 11.7	55.6 ± 12.3	0.031
Sex	Male	106 (64)	105 (64)	0.012
	Female	59 (36)	60 (36)	
Tumor location	Frontal	62 (38)	60 (35)	0.025
	Temporal	50 (30)	51 (31)	0.013
	Parietal	43 (26)	39 (24)	0.056
	Occipital	4 (2)	4 (2)	0.000
	Insula	2 (1)	2 (1)	0.000
	Thalamus	3 (2)	5 (3)	0.078
	Basal ganglia	1 (1)	4 (2)	0.149
Extent of resection	No residual lesion	105 (64)	106 (64)	0.012
	Unmeasurable residual lesion	25 (15)	26 (16)	0.016
	Measurable residual lesion	25 (15)	25 (15)	0.000
	Biopsy	10 (6)	8 (5)	0.053
KPS before adjuvant TMZ	100	24 (15)	24 (15)	0.000
	90	73 (44)	72 (44)	0.012
	80	33 (20)	37 (22)	0.059
	70	35 (21)	32 (19)	0.045
Japan Coma Scale before adjuvant TMZ	0	142 (86)	143 (87)	0.018
	1-3	23 (14)	22 (13)	
IDH gene	Wild-type	154 (93)	154 (93)	0.000
	Mutant	11 (7)	11 (7)	

Abbreviations: TTFields, tumor-treating fields; TMZ, temozolomide; KPS, Karnofsky Performance Status; IDH, isocitrate dehydrogenase

Supplemental Table 5. Five number summary of patinets' age between TTField users and nonusers in all patients and propensity score matched samples

	Minimum	25th percentile	Median	75th percentile	Maximum
All patients					
No TTFields	18	52	64	70	79
TTFields	25	45	56	64	79
Matched samples					
No TTFields	31	47	57	65.5	75
TTFields	29	47	57	65	75

Abbreviations: TTFields, tumor-treating fields
